# Reply to: Austin *et al*. CytoJournal 2009;6:12 (Unfounded claims mar scientific critique)

**DOI:** 10.4103/1742-6413.57780

**Published:** 2009-11-17

**Authors:** Jose Jeronimo, Mark A Barone, Silvana Luciani, Ricky Lu, Jacqueline Sherris, Julie Torod, Vivien Tsu

**Affiliations:** Address: Reproductive Health Global Program, PATH, Seattle, WA, USA; 1 EngenderHealth, New York, NY, USA; 2 Pan American Health Organization (PAHO), Washington, DC, USA; 3 Jhpeigo, Baltimore, MD, USA; 4 International Union Against Cancer (UICC), Geneva, Switzerland

To the Editor,

Austin and Zhao[1] raise accusations of anticytology bias and conflict of interest in the cervical cancer screening study published by Sankaranarayanan *et al.,*[2] which was very surprising as these were a repetition of the same previously published (and refuted) charges. The authors note that “questions have been raised” about the Alliance for Cervical Cancer Prevention (ACCP; see www.alliance-cxca.org) and its objectivity with regard to human papillomavirus (HPV) tests. They fail to acknowledge that all these questions have been repeatedly raised by the same person (lead author on all three references cited) and that they have also been answered in a peer-reviewed journal.[3] They also fail to acknowledge that the ACCP, for the past 8 years and in 20 countries, has assessed a variety of approaches to cervical cancer screening, including cytology.[4] Contrary to what is implied by Austin and Zhao, the ACCP—and, in particular, its coordinating organization, PATH—has not received any funding from the HPV test manufacturer for this or any other ACCP study. PATH has, in fact, been working with the manufacturer to develop a lower cost and simpler alternative to their current HPV test that would be more suitable for developing-country use. Unless the authors are charging that the supposed conflict of interest led to a flawed study design (in which case they should point out the flaw) or to deliberate data manipulation (a serious charge of scientific misconduct for which they must provide solid evidence), the claims simply cloud the scientific debate with unfounded accusations and do a disservice to readers.

We, as members of the ACCP, are committed to making screening and treatment technologies available that are feasible and effective in low-resource settings. If our pragmatic and evidence-based approach means that we are “enamored of the promise of science,” we have no difficulty with that charge. ACCP, without industry funding, continues to undertake a coordinated research agenda to assess cervical cancer screening and treatment approaches suited to low-resource settings, to improve service delivery systems, to ensure that community perspectives and needs are considered in programs, and to heighten awareness of cervical cancer prevention. We will continue to evaluate new data as it becomes available, but we will not sit by and watch women die needlessly when we can get started now with the tools we already have at hand and for which solid data exist—including visual inspection and, when less-expensive tests become available, HPV testing.

## COMPETING INTEREST STATEMENT BY ALL AUTHORS

No competing interest to declare by any of the authors.

## AUTHORSHIP STATEMENT BY ALL AUTHORS

All authors of this article declare that we qualify for authorship as defined by ICMJE http://www.icmje.org/#author.

Each author has participated sufficiently in the work and take public responsibility for appropriate portions of the content of this article.

Each author acknowledges that this final version was read and approved.

## Reply to: Jeronimo *et al*. CytoJournal 2009;6:23 (Unfounded claims mar scientific critique)

To the Editor,

Thank you for your interest in our commentary[5] on the article “HPV Screening for Cervical Cancer in Rural India”.[6,7] We note that the letter writers make no comment on the data-based questions raised by us and others[8–12] regarding the soundness of this study. For example, Suba *et al*. conclude, similar to us, that “unidentified differences in follow-up care, rather than differences among screening test detection rates, account for differences in mortality among the groups of women who underwent screening.”[8]

We also received unsolicited communications regarding our commentary from other Seattle-based investigators as follows – “We had an interesting journal club discussion. A summary of the major points:

1. The randomization process might not have worked because, overall, there were fewer cancers in the hc2 arm than in the other two intervention arms.
2. If hc2 screening prevented more deaths from cervical cancer than Pap or VIA, one would expect that it did so because it was more sensitive and allowed for early detection and treatment of cervical cancer and precancer. Thus, one would expect to find more screen-detected cancers and precancers in the hc2 arm than in the Pap and VIA arms, especially in a 30–59-year-old population with essentially no prior screening. However, as the prevalence of screen-detected CIN2-3 and cancer was similar for all three arms, screening test performance might not explain the observed intervention-related differences in cervical cancer mortality.
3. The lack of correlation between stage of cancer at time of diagnosis and subsequent death in the hc2 and Pap arms is perplexing.
4. The findings, overall (and with respect to point 3 above), might have been more interpretable had the postscreening clinical interventions (i.e., influence of HPV/Pap/VIA test results on number of biopsies, reading of biopsies, referral for treatment, adherence to treatment, recurrences, treatment of recurrences, etc.) been presented in greater detail. Differences in cancer deaths might have had more to do with differences in the diagnosis and treatment of lesions and cancers than with differences in screening test performance.”

We agree with the letter writers that women should not “die needlessly” of cervical cancer and continue to ponder the unanswered question of what was learnt in the Alliance for Cervical Cancer Prevention (ACCP)-supported study from inclusion of a controversial no-screening arm that one could not learn without it. This arm was associated with a substantial number of otherwise needless cervical cancer deaths.

Despite the apparent connections of commercial interests to the study, we certainly do not claim to have evidence that study irregularities were due to deliberate data manipulation. Readers, of course, will make their own personal judgements about study inconsistencies. Neither are we nor Suba *et al*. the only individuals to have raised questions about the policies of the Gates Foundation and studies conducted by the ACCP component organizations.[13] McCoy *et al.'s* recent Lancet article, for example, states that PATH “stands out” with its receipt of $1 billion dollars from the Gates Foundation and questions “whether some organizations might be better characterized as agents of the foundation rather than as independent grantees.” The Lancet article also questions the Gates Foundation's support of private sector development, stating that this “suggests that the Gates Foundation is keen to promote the growth of private health-care providers in low-income and middle-income countries.” The PATH website in turn refers to the manufacturer of the HPV test used in the NEJM study as one of its “private sector partners” in its START project.[14] One advantage of totally independent studies is that they do not have to overcome the additional burden associated with the numerous published observations that industry-linked studies tend to disproportionately reach conclusions favorable to industry and to use study designs likely to favor industry.[15]

## COMPETING INTEREST STATEMENT BY ALL AUTHORS

No competing interest to declare by any of the authors.

## AUTHORSHIP STATEMENT BY ALL AUTHORS

All authors of this article declare that we qualify for authorship as defined by ICMJE http://www.icmje.org/#author.

Each author has participated sufficiently in the work and take public responsibility for appropriate portions of the content of this article.

Each author acknowledges that this final version was read and approved.
